# Morphological neurite changes induced by porcupine inhibition are rescued by Wnt ligands

**DOI:** 10.1186/s12964-021-00709-y

**Published:** 2021-08-16

**Authors:** Juan A. Godoy, Jasson Espinoza-Caicedo, Nibaldo C. Inestrosa

**Affiliations:** 1 Centro de Envejecimiento y Regeneración (CARE-UC), Departamento de Biología Celular y Molecular, Facultad de Ciencias Biológicas, Pontificia Universidad Católica de Chile, Av. Libertador Bernardo O`Higgins 340, Santiago de Chile, Chile; 2 Centro de Excelencia en Biomedicina de Magallanes (CEBIMA), Universidad de Magallanes, Punta Arenas, Chile

**Keywords:** Wnt signaling, Porcupine, Neurites, Dendritic arbor complexity, Embryonic hippocampal neurons

## Abstract

**Background:**

Wnt signaling plays key roles in cellular and physiological processes, including cell proliferation, differentiation and migration during development and tissue homeostasis in adults. This pathway can be defined as Wnt/β-catenin-dependent or β-catenin-independent or “non-canonical”, both signaling are involved in neurite and synapse development/maintenance. Porcupine (PORCN), an acylase that o-acylates Wnt ligands, a major modification in secretion and interaction with its receptors. We use Wnt-C59, a specific PORCN inhibitor, to block the secretion of endogenous Wnts in embryonic hippocampal neurons (DIV 4). Under these conditions, the activity of exogenous Wnt ligands on the complexity of the dendritic tree and axonal polarity were evaluated

**Methods:**

Cultured primary embryonic hippocampal neurons obtained from Sprague–Dawley rat fetuses (E18), were cultured until day in vitro (DIV) 4 (according to Banker´s protocol) and treated with Wnt-C59 for 24 h, Wnt ligands were added to the cultures on DIV 3 for 24 h. Dendritic arbors and neurites were analysis by fluorescence microscopy. Transfection with Lipofectamine 2000 on DIV 2 of plasmid expressing eGFP and KIF5-Cherry was carried out to evaluate neuronal polarity. Immunostaining was performed with MAP1B and Tau protein. Immunoblot analysis was carried out with Wnt3a, β-catenin and GSK-3β (p-Ser9). Quantitative analysis of dendrite morphology was carried out with ImageJ (NIH) software with Neuron J Plugin.

**Results:**

We report, here, that Wnt-C59 treatment changed the morphology of the dendritic arbors and neurites of embryonic hippocampal neurons, with decreases β-catenin and Wnt3a and an apparent increase in GSK-3β (p-Ser9) levels. No effect was observed on axonal polarity. In sister cultures, addition of exogenous Wnt3a, 5a and 7a ligands rescued the changes in neuronal morphology. Wnt3a restored the length of neurites to near that of the control, but Wnt7a increased the neurite length beyond that of the control. Wnt5a also restored the length of neurites relative to Wnt concentrations.

**Conclusions:**

Results indicated that Wnt ligands, added exogenously, restored dendritic arbor complexity in embryonic hippocampal neurons, previously treated with a high affinity specific Porcupine inhibitor. We proposed that PORCN is an emerging molecular target of interest in the search for preclinical options to study and treat Wnt-related diseases.

**Video Abstract**

**Supplementary Information:**

The online version contains supplementary material available at 10.1186/s12964-021-00709-y.

## Background

Proper functioning of the Wnt signaling pathway is critical to the regulation of numerous processes, including cellular polarity, cell proliferation and cellular differentiation [1, 2]. Wnt ligands are growth factors that can influence the cell cycle and affect cell proliferation; however, they also contribute to cytoskeleton arrangement and therefore give directionality to cell proliferation, regulate spatial growth and provide signals to orient asymmetric stem cell division in vitro [3–5]. The Wnt signaling pathway is active in various types of cells in the central nervous system (CNS), including fibroblast-like cells, radial glia, oligodendrocytes, microglia/macrophages, astrocytes and neurons [6–9]. In the CNS, Wnt signaling, also participates in the formation of synapses and neuronal circuits, and in the adult brain, Wnt signaling modulates synaptic transmission, plasticity and neurogenesis [6, 10–12]. Wnt ligands are secreted short-range molecules that can trigger two intracellular pathways, the Wnt-β-catenin-dependent and the "noncanonical" β-catenin-independent signaling [13, 14]. Nineteen Wnt proteins have been described in humans and recognized by the Frizzled (Fzd) family of receptors [2].

Neurons are highly polarized cells with two distinct morphological structures, axons and dendrites. Synapses form between the contacts of these two specialized regions. In general, the axon is a long process that communicates with other neurons through the release of neurotransmitters [15, 16]. Dendrites are more complex structures formed by multiple branching processes and dendritic spines, which are small protuberance of the plasma membrane where the postsynaptic excitatory apparatus is assembled and are essential for the regulation of postsynaptic receptor levels [17, 18]. The formation and complexity of these processes is critical to the directional flow of information between neurons in the CNS. Granular cerebellar, cortical, and hippocampal neurons become polarized as they differentiate, and a cell culture system was established to study rodent hippocampal neurons in vitro more than four decades ago. This in vitro system has been fundamental in research on the structuring of neuronal shape and cellular and molecular mechanisms during neuronal polarization [19, 20]. In that in vitro model, hippocampal neurons spontaneously polarize, spreading filopodia, and lamellipodia appear with small neurites, then one of the neurites grows faster to become an axon, and the remaining neurites develop into dendrites. Finally, in later stages of culture, axons and dendrites undergo further development to form functional synapses [21, 22].

The aim of our work was to evaluate the effect of the activity of the Wnt ligands, in embryonic neurons previously treated with a high affinity specific inhibitor Wnt-C59 of porcupine [23], a membrane-bound O-acyltransferase required for the palmitoylation, secretion and biological activity of Wnts [24–27]. Rescue experiments with specific Wnt ligands were carried out to determine the contribution of individual Wnt ligands to dendritic arbor formation and the maintenance of dendritic spines.

## Methods

### Animals and ethical standards

Experiments were performed on Sprague–Dawley rat fetuses (E18). All rats were housed at the Animal Warehouse Facility of the Pontificia Universidad Católica de Chile. The Bioethical and Biosafety Committee of the Faculty of Biological Sciences of our university approved the experimental procedures.

### Primary rat hippocampal neuron cultures

Hippocampal neurons were obtained from 18-day-old Sprague–Dawley rat embryos [19]. Hippocampi were aseptically dissected and trypsinized for 16 min. After centrifugation for 1 min, cells were seeded in phenol red-free Dulbecco's modified Eagle's medium plus 10% horse serum into 1% poly-L-lysine-coated plates. After 120 min, the medium was removed, and neurobasal medium containing 1% B27 supplement (Invitrogen), streptomycin and penicillin was added. To obtain embryonic hippocampal neurons, neurons were cultured until day in vitro (DIV) 4 and treated with different concentrations of Wnt-C59 for 24 h, and different Wnt ligands were added to the cultures on DIV 3 for 24 h.

### Reagents

Antibodies against ATF2, MAP1B, Tau, Ankirin G, β-catenin, GSK-3 β(ser9), β-actin, MAP2 and phalloidin were obtained from Cell Signaling Technology, Inc. (Trask Lane, Danvers, MA). Neurobasal growth medium was obtained from Life Technologies (Carlsbad, CA). Other reagents were purchased from Sigma (St. Louis, MO). Wnt-C59 inhibitor was dissolved in DMSO and added to neuronal cultures at a final concentration of 0.01% DMSO or less (Cellagen Technology, San Diego, CA). Recombinant Wnt3a, Wnt5a, and Wnt7a (R&D Systems, Abingdon, UK) were used at 50 ng/ml, 100 ng/ml, and 200 ng/ml, respectively with corresponding dilutions in vehicle.

### Fluorescence microscopy

After fixation, neurons were permeabilized with 0.5% (v/v) Triton X-100 in 1 × PBS for 5 min and washed in 1 × in PBS. Then, the cells were incubated for 30 min in blocking solution (2% glycine, 2% BSA, 5% FBS). To study dendrite morphology neurons were stained with MAP2 and Tau antibodies. Highly cross-absorbed Alexa-conjugated antibodies were used as secondary labels. Coverslips were mounted on slides with Vectashield (Vector Labs) medium and images were captured in a Carl Zeiss LSM microscope.

### Transfection of neuronal hippocampal cultures

Primary hippocampal neuronal cultures were transfected with Lipofectamine 2000 on DIV 2 with plasmid expressing eGFP or KIF5-cherry, and cells were fixed on DIV 4. Immunostaining was performed for MAP1B and Tau protein. Neuronal cultures were fixed at the times indicated in the figure legends with LF fixative solution (60 mM PIPES, 25 mM HEPES, 10 mM EDTA, 2 mM MgCl2, 0.12 M sucrose, 4% paraformaldehyde) for 5 min and then washed once with phosphate buffered saline (PBS) before processing for immunostaining.

### Immunoblot analysis

Total protein extraction from cultured hippocampal neurons and immunoblot analysis were performed as previously described [28]. At the designated times, cells were lysed in RIPA buffer (10 mM Tris–HCl, pH 7.4, 5 mM EDTA, 1% NP-40, 1% sodium deoxycholate, and 1% SDS) supplemented with a protease inhibitor mixture (1 mM PMSF, 2 μg/mL aprotinin, 1 μg/mL pepstatin and 10 μg/mL benzamidine) and phosphatase inhibitors (25 mM NaF, 100 mM Na_3_VO_4_, 1 mM EDTA and 30 μM Na_4_P_2_O_7_), homogenized by scraping, and then passed sequentially through different caliber syringes. Protein samples were centrifuged twice at 10,000 rpm and 4 °C for 5 min. Protein concentration was determined using the BCA Protein Assay Kit (Pierce). Samples were resolved by 10% SDS-PAGE and transferred to a PVDF membrane. The membranes were incubated with antibodies against β-catenin, GSK-3 β(p-ser9), Wnt3a (Thermo Scientific, Rockford, IL), (Cell Signaling Technology, Danvers, MA) and β-actin (Sigma-Aldrich, St. Louis, MO), followed by anti-mouse, anti-goat or anti-rabbit IgG peroxidase-conjugated antibodies (Pierce, Rockford, IL). The membranes were developed using an ECL Kit (Western Lightning Plus ECL, PerkinElmer).

### Image and statistical analysis

The quantitative analysis of dendrite morphology was carried out with ImageJ (NIH) software with the assistance of the Neuron J Plugin. All dendrites from each neuron were quantified for parameters such as length and primary, secondary and tertiary dendrite numbers. All data are expressed as the mean ± SEM, and the number of experiments is indicated in the corresponding figures. Differences between groups were determined by ANOVA, one-way post hoc Bonferroni of multiple comparisons to establish significant differences. A value of *p* < 0.05 was considered significant.

## Results

### Inhibition of porcupine by Wnt-C59 triggers the loss of neurites in embryonic cultures of hippocampal neurons

**Fig. 1:**
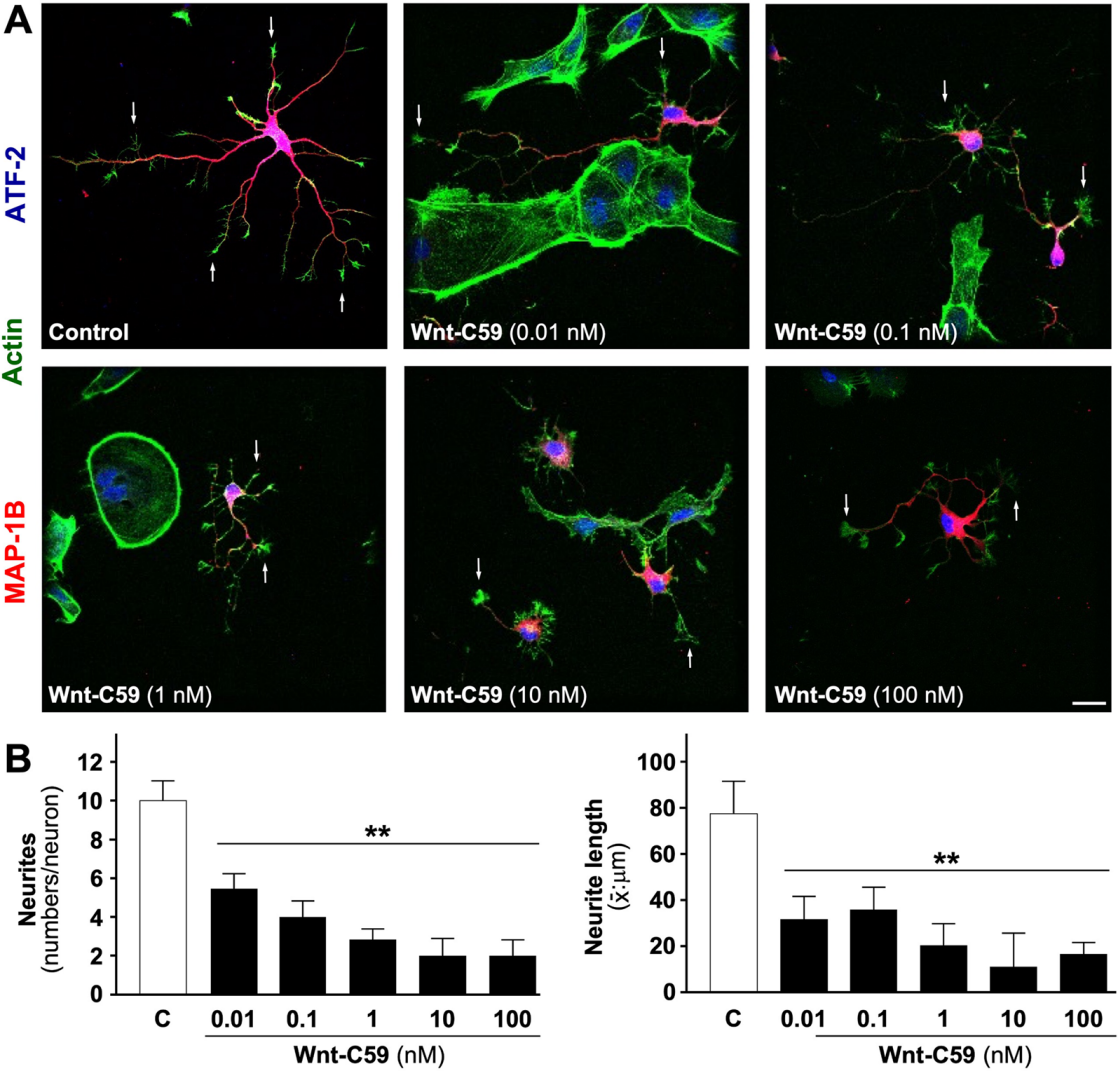
A Porcupine inhibitor, Wnt-C59 induces morphological change in dendritic arbor. Hippocampal neurons were cultured until 4DIV and treated with different concentration of Wnt-C59 (0.01, 0.1, 1, 10, 100 nM) for 36 h, and stained with MAP1B (red), ATF-2 (blue) and F-actin marker phalloidin (green). Neurons were imaged using a confocal microscope to evaluate neuronal morphology. **A** Representative Micrographs are showed, Wnt-C59 produces a decrease of the dendritic arbor in these embryonic neurons; Interestingly, a large amount of terminal filopodia/lamellipodia was observed with Wnt-C59 treatment (**A**, see white arrow). **B** The quantification of these processes is shows as representative data from embryonic cultured neurons. The results are presented as the mean ± SEM for n: 10–12 experiments), **p < 0.01. Bonferroni’s test. Bar represents 10 μm

Neurons construct a characteristic polarized dendrite axis, which is physiologically important for receiving and processing axon synaptic signals. In low-density cultures, polarization and active axon growth occur between DIV 2–6; therefore, this time is ideal to study the role of endogenous Wnt signaling in dendritic tree formation and axonal processes. For this purpose, we blocked Wnt signaling with the acylase inhibitor Wnt-C59 which is specific for the acylase PORCN. Rat hippocampal neurons DIV 4, were treated with different Wnt-C59 concentrations (0, 0.1, 1, 10, and 100 nM) for 36 h, and stained for microtubule-associated protein 1B (MAP1B) which is abundantly expressed in actively spreading neurons [29]. Neurites were visualized with phalloidin which labels F-actin and nuclei were stained with ATF-2. Our results showed that PORCN inhibition significantly decreased both the number of neurites/neurons (Fig. 1A; see representative micrographs, **p < 0.01) and the length of the neurites (Fig. 1B, **p < 0.01). Interestingly, a large amount of terminal filopodia/lamellipodia was observed with Wnt-C59 treatment (Fig. 1A, see white arrow).

### Inhibition of porcupine by Wnt-C59 results in loss of contact with other cells in embryonic cultures of hippocampal neurons

**Fig. 2:**
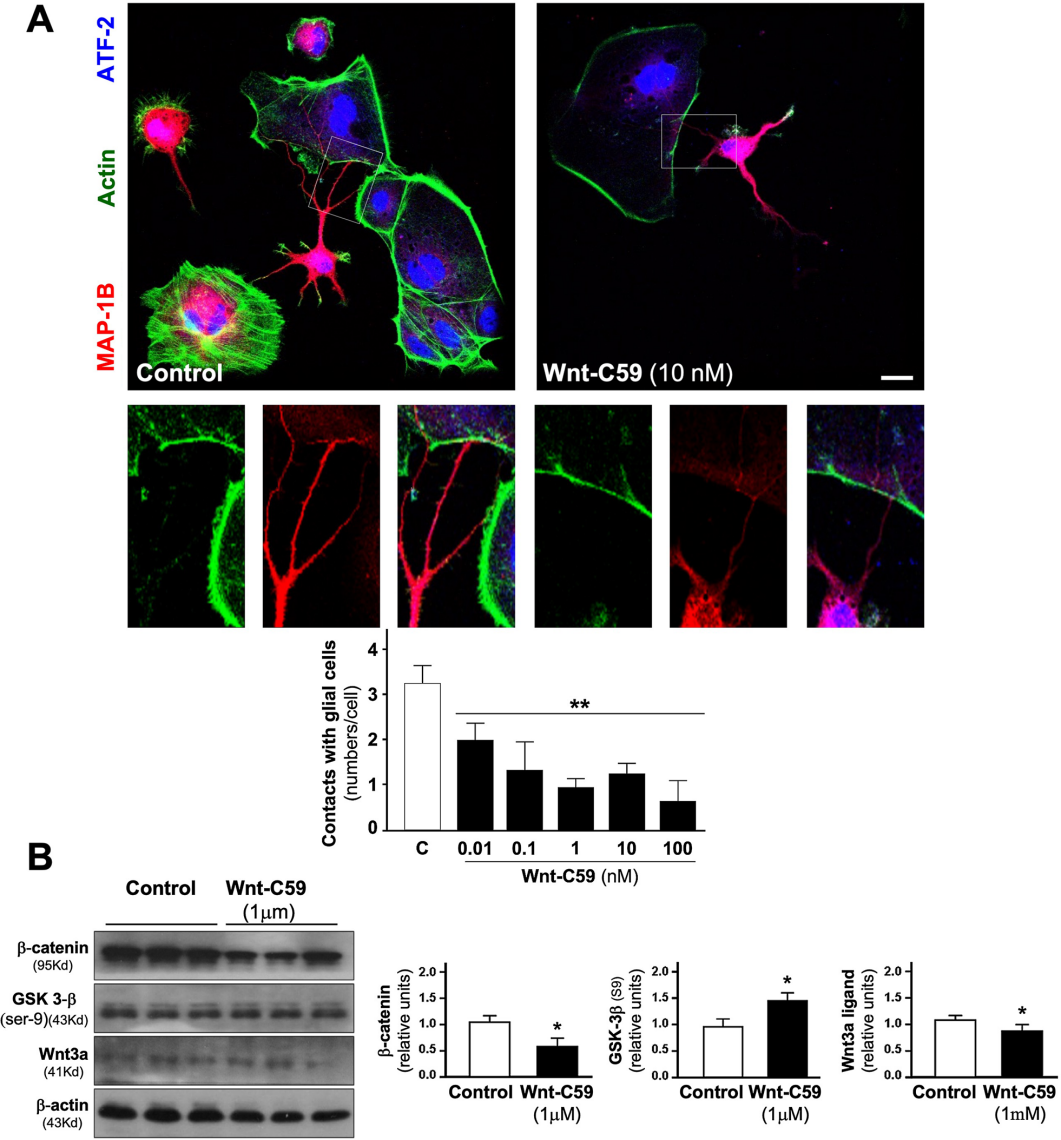
A Porcupine inhibitor, Wnt-C59, produces a loss of contacts with other types of cells in culture. **A** Sister cultures of hippocampal neurons were used to quantify the contact of neurons with other cell types, without specifying the cell type; these contacts of neuronal processes decrease by half, depending also on the concentration of the inhibitor. See representative micrographs of control neurons and treated with 10 nM Wnt-C59 for 24 h. and zoom. **B** The figure shows a representative blot of some proteins of the Wnt pathway, under the inhibitory activity of Wnt-C59 in hippocampal neurons. Graph shows quantification of these contacts. The results are presented as the mean ± SEM for n = 10 experiments), *p < 0.05, **p < 0.01. Bonferroni’s test. Bar represents 10 μm

Another phenomenon that was verified in our experiments is the loss of contacts with other types of cells present in primary cultures. This phenomenon was evaluated by counting the number of neuronal processes in contact with another type of nonneuronal cell. The number of contacts with another cell type decreases by half when the neuronal cultures are treated with the Wnt-C59 inhibitor (Fig. 2A, see representative micrographs and lower graph, **p < 0.01). The treatment of hippocampal neuron cultures with the inhibitor also results in a significant decrease in β-catenin, a key protein in the Wnt signaling pathway, with an apparent increase in the inactive form of GSK-3β kinase (phosphorylated in serine 9). The Wnt3a ligand also decreased after Wnt-C59 treatment (Fig. 2B, see representative blot and quantification of different proteins, *p < 0.05). See Additional file 1: Fig. S1, for further studies on the changes in β-catenin and Wnt3a in L cells.

### Inhibition of porcupine by Wnt-C59 treatment does not change axonal polarity in embryonic hippocampal neurons

**Fig. 3:**
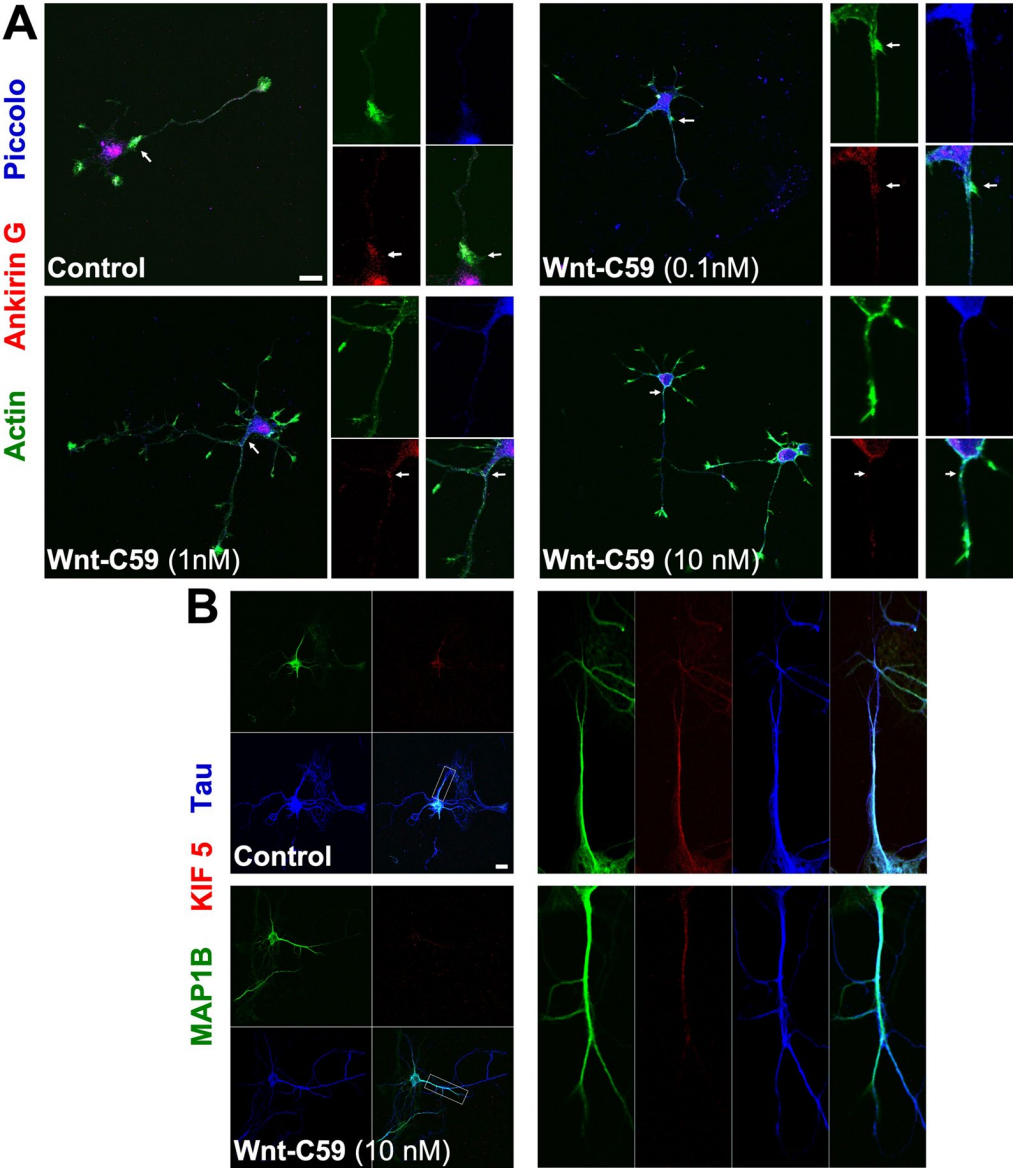
Porcupine inhibition by Wnt-C59 treatments does not change the axon polarity in embryonic hippocampal neurons. Neurons (DIV 4) were treated with different concentration of Wnt-C59 (0.1, 1, 10 nM) for 24 h. **A** AnkG protein (red), Piccolo a protein present in the Piccolo-Bassoon transport vesicle (PTV) (blue) and actin (green) were stained to locate AIS (see white arrow). **B** Sister cultured were transfected with KIF 5-Cherry and with MAP1b and Tau protein, to locate AIS in axonal processes (see zoom of label region in axonal region). Bar represents 10 μm

The polarized sorting and trafficking of newly synthesized proteins to the somato-dendritic and axonal domains of neurons occurs by selective incorporation into distinct populations of vesicular transport carriers. Voltage-gated Na^+^ and K^+^ channels are crucial for the efficient initiation and saltatory propagation of action potentials along myelinated axons of vertebrates, are clustered at the axon initial segment (AIS) and nodes of Ranvier. The clustering of these channels is mediated by ankyrin-G (AnkG) [30]. Previous studies concluded that AIS, an actin-based filter, selectively prevents the passage of somato-dendritic vesicles into the axon [31]. Conventional kinesin-1 is a major anterograde motor that operates in axons and consists of a heavy-chain (KIF5A, KIF5B, or KIF5C) dimer and two light chains (KLC) binding to the C termini of the dimer [32]. One of the questions that arises from our experiments is whether the inhibition of PORCN, with the consequent lack of ligands and activation of the Wnt pathway, might alter neuronal polarity in some way. For this purpose, embryonic neurons were treated with the inhibitor Wnt-C59 and two proteins of the AIS were studied, AnkG and KIF5 [31, 33]. Our results showed that both proteins were correctly located in the neuronal axon of the neuron (Fig. 3). Embryonic neurons were treated with different concentrations of Wnt-C59 (0.1, 1, 10 nM) and stained for AnkG and Piccolo, a protein present in the Piccolo-Bassoon transport vesicle (PTV), which are dense particles that form the active zone of the neurons [15]. Our experiments show that both proteins are correctly located, together with actin activity in the same place (Fig. 3A, see representative micrograph, white arrow shows AIS). Embryonic hippocampal neurons transfected with KIF5 and treated with 10 nM Wnt-C59, showed a similar localization to control cells together with specific proteins of the neuronal axon (Fig. 3B, see representative micrographs). We conclude that inhibition of porcupine with Wnt-C59 did not affect the neuronal polarity of the axon.

### Different exogenous Wnt ligands recover the morphology of embryonic hippocampal neurons in culture

To determine the specific role of specific Wnt ligands, embryonic primary hippocampal neurons were treated with Wnt-C59 to shut down Wnt signaling activity. We evaluated the individual activity of Wnt ligands with canonical activity, such as Wnt 3a and 7a, and a known non canonical Wnt5a ligand.

#### Treatment with exogenous canonical Wnt3a ligand on neuronal morphology

**Fig. 4:**
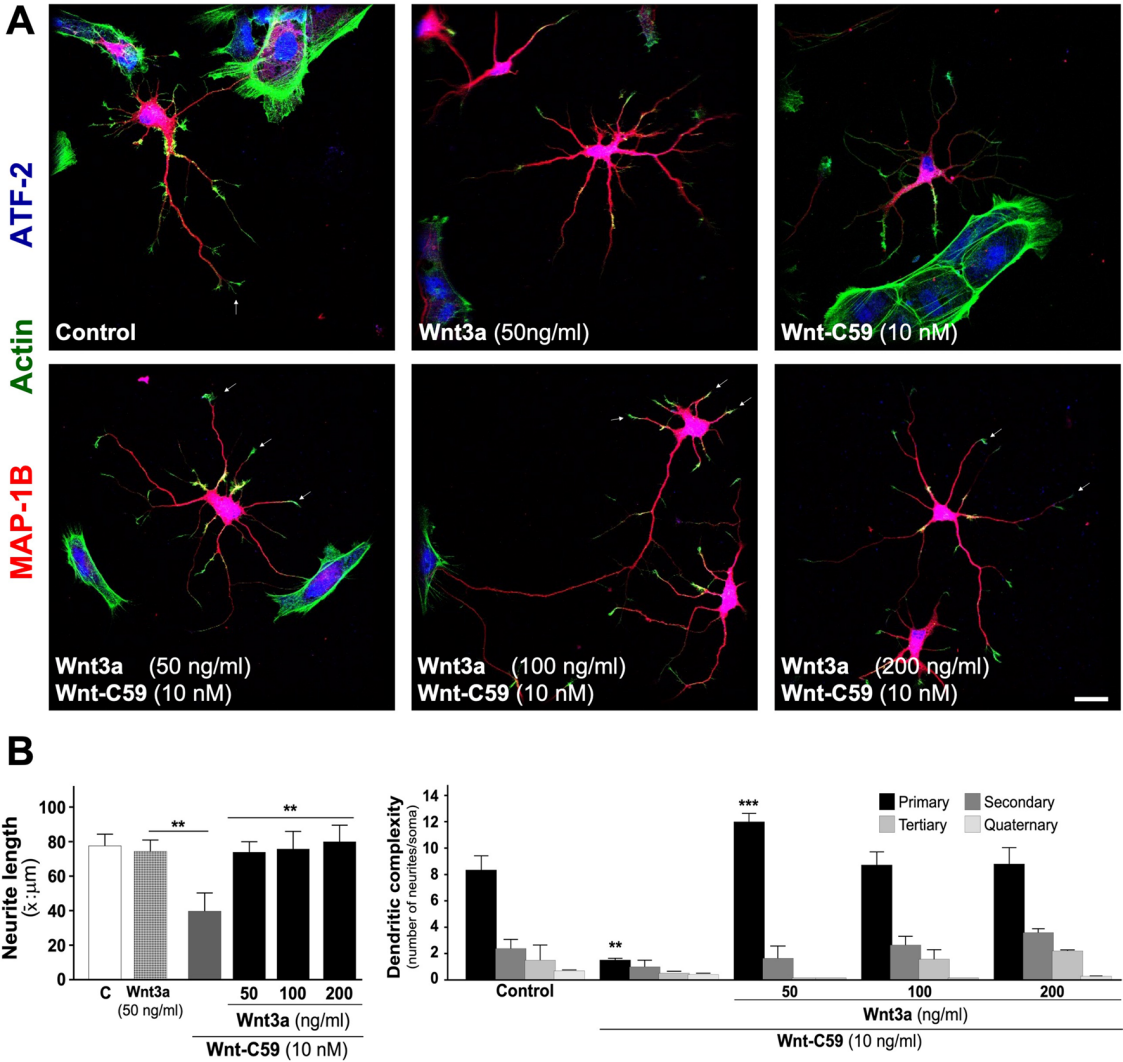
Exogenous Wnt3a ligand treatment recovery dendritic arbor after inhibition of PORCN. Sister cultures of embryonic hippocampal neurons were used to quantify the recovery of dendritic arbor after treatment with different concentrations of the canonical Wnt3a ligand. **A** See representative micrographs of control neurons and treated with Wnt3a (50 ng/ml), 10 nM Wnt-C59 or plus Wnt3a, (50, 100, 200 ng/ml) for 24 h, White arrowhead show actin on the tip of different neurites. **B** Graph shows quantification of average of neurites length (see left graph) and recovery of dendritic complexity (see right graph). The results are presented as the mean ± SEM for n = 5 experiments, **p < 0.01, ***p < 0.001. Bonferroni’s test. Bar represents 10 μm

Embryonic hippocampal neurons (DIV 4), were treated with 10 nM Wnt-C59 from DIV 2 to DIV 4. These cultures were treated with different concentrations of the Wnt3a ligand for 24 h (50, 100, 200 ng/ml) and stained with MAP-1B (red), ATF-2 a nuclear transcription factor (blue) and phalloidin to observe actin (green), which can be seen at the tips of neuronal processes and to a lesser extent in neurons treated with Wnt-C59 (see white arrows). Embryonic neurons treated with Wnt3a showed a recovery of the length of neurites similar to the control (Fig. 4A, see representative micrographs). Another parameter that was recovered was the dendritic arbor complexity, concomitant with the recovery of secondary and tertiary projections (Fig. 4B, see graphs).

#### Treatment with exogenous noncanonical Wnt5a ligand on neuronal morphology

**Fig. 5:**
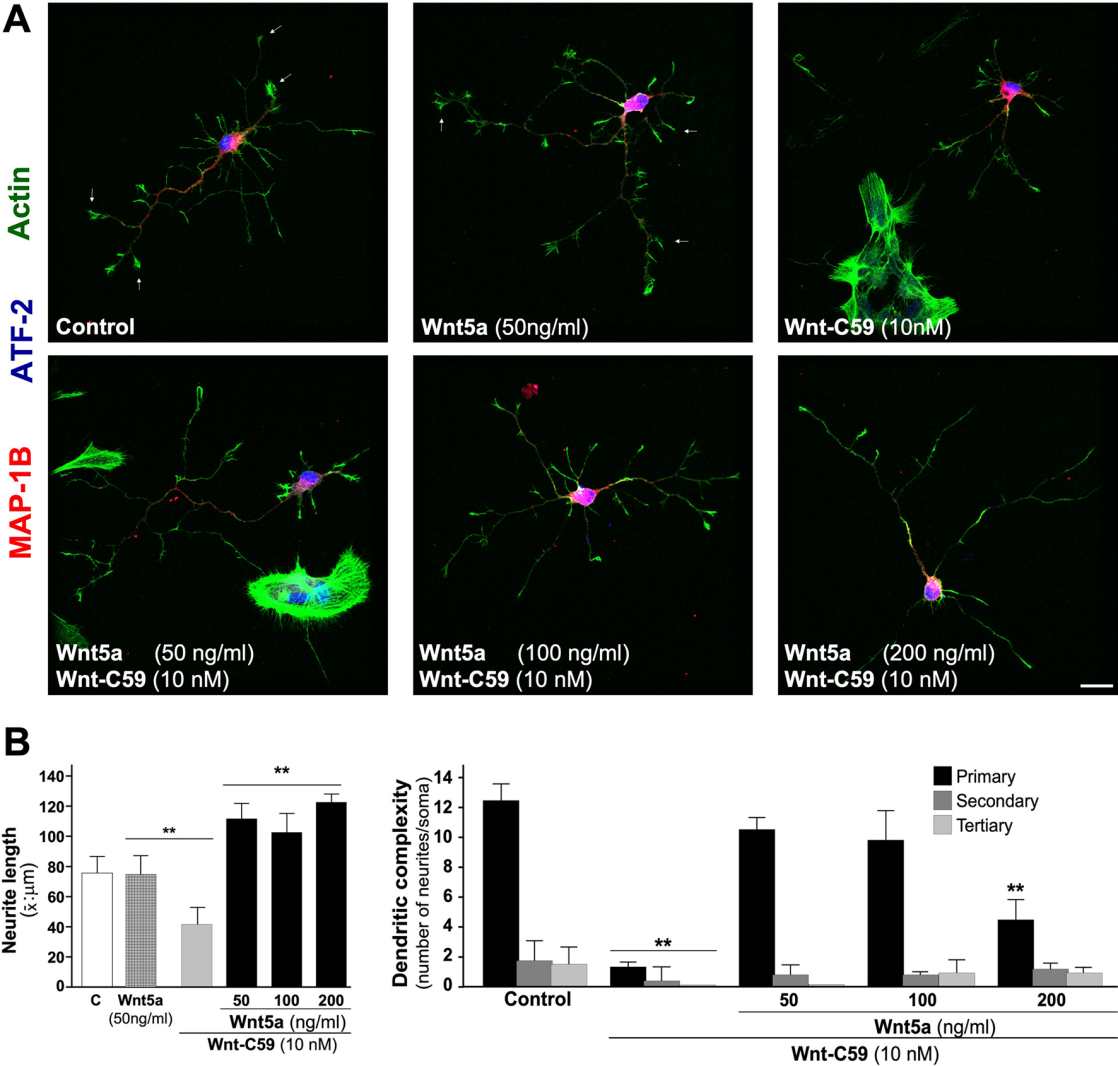
Exogenous Wnt5a ligand treatment recovers dendritic arbor after inhibition of PORCN. Sister cultures of embryonic hippocampal neurons were used to quantify the recovery of dendritic arbor, after treatment with different concentrations of the non-canonical Wnt5a ligand. **A** See representative micrographs of control neurons and treated with Wnt5a (50 ng/ml), 10 nM Wnt-C59 or plus Wnt5a, (50, 100, 200 ng/ml) for 24 h, White arrowhead show actin on the tip of different neurites. **B** Graph shows quantification of average of neurite length (see left graph) and recovery of dendritic complexity (see right graph). The results are presented as the mean ± SEM for n = 5 experiments, **p < 0.01. Bonferroni’s test. Bar represents 10 μm

Sister embryonic hippocampal neurons (DIV 4), were treated with 10 nM Wnt-C59 from DIV 2 to DIV 4. These cultures were treated with different concentrations of Wnt5a for 24 h (50, 100, 200 ng/ml) and stained with MAP1B (red), ATF (blue) and phalloidin to visualize actin (green), which can be observed at the tips of neuronal processes and to a lesser extent in neurons treated with Wnt-C59 (Fig. 5; representative micrographs). Our results showed that embryonic neurons treated with different concentrations of Wnt5a showed a recovery of the length of neurites to a longer extension than control neurons, by 40–50% of the control depending on Wnt5a concentration (Fig. 5A, see representative micrographs) F-actin activity was marked with phalloidin (see white arrow). The complexity of the dendritic arbor was also recovered, together with the secondary and tertiary projections (Fig. 5B, see graph).

#### Effect of treatment with exogenous canonical Wnt7a ligand on neuronal morphology

**Fig. 6:**
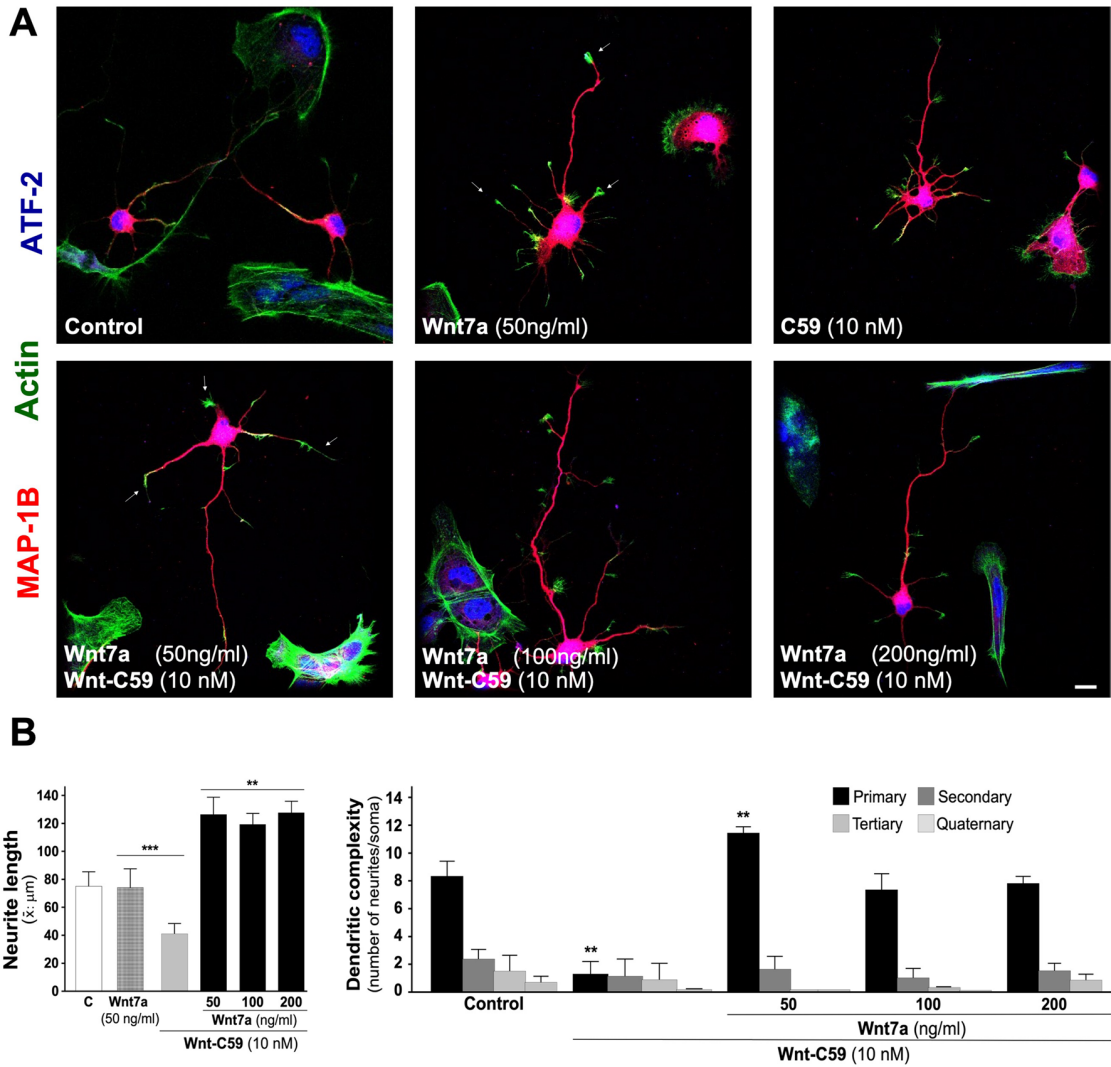
Exogenous Wnt7a ligand treatment recovery dendritic arbor after inhibition of PORCN. Sister cultures of embryonic hippocampal neurons were used to quantify the recovery of dendritic arbor after treatment with different concentrations of the canonical Wnt7a ligand, and stained with MAP1B (red), ATF-2 (blue) and phalloidin (green). Neurons were imaged using a confocal microscope to evaluate neuronal morphology. **A** See representative micrographs of control neurons and treated with Wnt7a (50 ng/ml), 10 nM Wnt-C59 or plus Wnt7a (50, 100, 200 ng/ml) for 24 h, White arrowhead show actin on the tip of different neurites. **B** Graph shows quantification of average of neurites length (see left graph) and recovery of dendritic complexity (see right graph). The results are presented as the mean ± SEM for n = 5 experiments, **p < 0.01, ***p < 0.001. Bonferroni’s test. Bar represents 10 μm

Embryonic hippocampal neurons (DIV 4), were treated with 10 nM Wnt-C59 from DIV 2 to DIV 4. These cultures were treated with different concentrations of the Wnt7a ligand for 24 h (50, 100, 200 ng/ml) and stained with MAP-1B (red), ATF-2 a nuclear transcription factor (blue) and phalloidin to visualize actin (green), which can be observed at the tips of neuronal processes (see white arrow). The embryonic neurons treated with Wnt7a ligand showed a recovery of the length of neurites to a much longer extension than the control, but at a much lower concentration of Wnt7a than of Wnt5a (Fig. 6A, see representative micrographs; B, left graph). According to our results, 24-h treatment of embryonic neurons restores dendritic tree complexity, to an extent similar to that observed with the control (Fig. 6B, see right graph).

## Discussion

Wnt signaling acts at various stages during neural development, including neuronal migration, axon and dendrite development, synapse formation/maturation, and neuronal plasticity [2, 6, 34]. Wnt pathways are classified into canonical Wnt/β-catenin or noncanonical (β-catenin-independent) pathways. Canonical Wnt/β-catenin signaling is mediated by nuclear translocation of its central effector β-catenin. Noncanonical Wnt signaling occurs independently of β-catenin and is stimulated by Wnt ligands that bind to a receptor complex of Fzd, Ror1/2 or Ryk [1, 13, 35].

A series of small molecule inhibitors specifically target several of the proteins in the Wnt signaling pathway, such as Fzd, DVL, PORCN or Tankyrase, and could also be used as chemical probes to dissect the mechanism(s) of these signaling pathways [36, 37]. We therefore used Wnt-C59, a PORCN inhibitor, in embryonic hippocampal neurons, to research polarization, the development of dendritic trees, and maintenance and/or maturation of dendritic spines; these inhibitory molecules have the potential to be developed both as study reagents in cell biology as therapeutic agents [38, 39]. Our present results show that the early inhibition of PORCN in embryonic hippocampal neurons, starting at picomolar Wnt-C59 concentration, drastically reduces the length of the neurites by up to 80%, and affects the dendritic arbor complexity and the signaling and contact that could be established between the different cells in culture. Nonneuronal cells are known to show no contact inhibition when in contact with the growth cones or neurites of neurons. These observations on the contact behavior of nonneuronal cells imply a series of signals from both types of cells, which in our experiments are lost with the inhibition of the Wnt pathway [40]. Together, these results, show that the endogenous production of the different Wnt ligands plays an early and important role in the growth and architecture of neurites and the signaling between the different cells of the in vitro neuronal culture.

To evaluate the individual activity of Wnt ligands, sister cultures of hippocampal neurons previously treated with Wnt-C59 to shut down Wnt signaling activity were incubated with exogenous Wnts. First, we tested Wnt3a, a ligand that has canonical activity but has also shown some Ca^2+^ modulation [41, 42]. Then, tested a noncanonical ligand Wnt5a which stimulates PSD-95 clustering [43] and finally, Wnt7a, a classic ligand of the Wnt-β-catenin-dependent pathway, which promotes the presynaptic colocalization of several neural receptors [34, 44]. Our results indicated that all exogenous Wnt ligands (Wnt3a, 5a and 7a) rescued the changes in neuronal morphology. Specifically, Wnt3a restores the length of neurites to a value similar to that of the control; however, Wnt7a increased the neurite length beyond that of the control. Wnt5a had the same effect but at higher relative Wnt concentration. In addition, all 3 Wnts ligands restored dendritic arbor complexity with the recovery of secondary and tertiary projections.

Previous studies have indicated that specific Wnt ligands induce neurite extension in various neuronal types: Wnt3 and Wnt3a in cultures of neural precursors of spinal cord cells [45], Wnt7a in cultured mouse cerebellar granule cells [46], Wnt5a and Wnt3b in chick retinal ganglion cell axons [47] and Wnt7b in mouse hippocampal neurons [48]. More interestingly, different Wnt ligands have distinct effects on growing axons, and canonical and noncanonical pathways can have opposite effects [2]. Adding to the complexity, the canonical ligand Wnt3a can activate both canonical and noncanonical signaling pathways in the same cell type [13, 49]. Although the interaction between canonical and noncanonical Wnt signaling is complex, these signaling pathways generally inhibit each, as demonstrated during development [50].

Recent studies in our laboratory with Wnt-C59, a specific PORCN inhibitor in SH-SY5Y cells, indicated that both the canonical Wnt3a ligand and a noncanonical Wnt5a ligand increased the basal expression of Teneurin [51], a new trans-synaptic signal in both the peripheral nervous system and the CNS [52, 53]. This work not only demonstrate the involvement of Wnt signaling in regulating Ten-3 expression but also revealed that Wnt3a (a canonical Wnt ligand) increases the expression of Ten-3 through a mechanism dependent on the secretion and activity of Wnt5a (a noncanonical ligand). Although the work raises new questions, the results seem to demonstrate the upregulation of Ten-3 by Wnt signaling and suggest that Ten-3 modulation is possible because of crosstalk between the canonical and noncanonical Wnt pathways [51].

An alternative derived from our results is the possibility of modulating the neuronal morphology in the neurogenic niche and in a determined time frame, after PORCN inhibition by Wnt-C59, using the “replacement” of specific Wnt ligands, and thereby avoiding other secreted endogenous Wnt inhibitors, such as Dickkopf-1 (Dkk-1) or the secreted-frizzled-related protein (sFRP), which would allow the formation of new connections and therefore new neural networks [38, 54, 55]. An interesting example is Wnt signaling inhibition by Wnt-C59 which prevents the activity of Wnt3a and Wnt8B, in canonical and noncanonical Wnt-mediated signaling to induce cortical motor neurons or their progenitors from iPSCs in humans [26, 56]. This strategy might offer a proof of concept on the preclinical side, where the inhibition of Wnts signaling in mammals can be achieved by inhibiting PORCN with small molecule inhibitors such as Wnt-C59, providing a safe and feasible strategy in vivo. Our results increase the possibility that a specific PORCN inhibitor contributes to regulating the Wnt pathway so that it become a safe and plausible methodology for cell replacement therapy in neurological diseases.

## Conclusions

Our studies showed that several Wnt ligands, including Wnt3a, Wnt7a and Wnt5a, exogenously aggregated, are able to overcome altered dendritic arbor complexity with recovery of secondary and tertiary projections in hippocampal neurons pre-treated with a specific PORCN inhibitor. We suggest that PORCN in Wnt signaling is a potential molecular target in the search for preclinical options to study and treat Wnt-related diseases.

## Supplementary Information

Additional file 1: Fig. S1. Wnt-C59 prevents accumulation of β-Catenin and increase Wnt3a in L Cells. **A**. Representative Western blot of β-Catenin levels in L cells that stably express Wnt3a, treated with Wnt-C59 (1 nM and 10 nM C59) for 48 hours. Quantification of β-Catenin protein levels after treatment with Wnt-C59. All data are presented as means ± SEM, it was performed as a two-way ANOVA statistical analysis n = 3. *p <0.05. ** p <0.01. **B**. Representative Western blot of Wnt3A levels in L cells treated with Wnt-C59 (1 nM and 10 nM) for 48 hours. Quantification of Wnt-3A protein levels after treatment with Wnt-C59 for 48 hours. All data are presented as means ± SEM, it was performed as a two-way ANOVA statistical analysis n = 3. *p <0.05. ** p <0.01.

## Data Availability

The datasets used and/or analyzed during the current study are available from the corresponding author on reasonable request.

## Abbreviations

AIS: Axon initial segment
ANOVA: Analysis of variance
ATF2: Activating transcription factor 2
CNS: Central nervous system
Dkk-1: Dickkopf-1
DIV: Days in vitro
DVL: Dishevelled
ECL: Enhanced chemiluminescence
EDTA: Ethylenediaminetetraacetic acid
eGFP: Enhanced Green Fluorescent Protein
Fzd: Frizzled
GSK-3β: Glycogen synthase kinase-3β
MAP1B: Microtubule-associated protein 1B
NaF: Sodium fluoride
PSD-95: Postsynaptic density protein 95
PTV: Piccolo-Bassoon transport vesicle
PVDF: Polyvinylidene difluoride
PORCN: Porcupine
sFRP: Secreted-frizzled-related protein
SDS-PAGE: Sodium dodecyl sulfate polyacrylamide gel electrophoresis
SEM: Standard error of the mean
RIPA: Radioimmunoprecipitation assay buffer
Wnt: Wingless
