# Supplementary figures and images for: Morphological neurite changes induced by porcupine inhibition are rescued by Wnt ligands
Source: Cell Commun Signal. 2021 Aug 16;19:87. doi: 10.1186/s12964-021-00709-y (PMC8369806; doi:10.1186/s12964-021-00709-y)

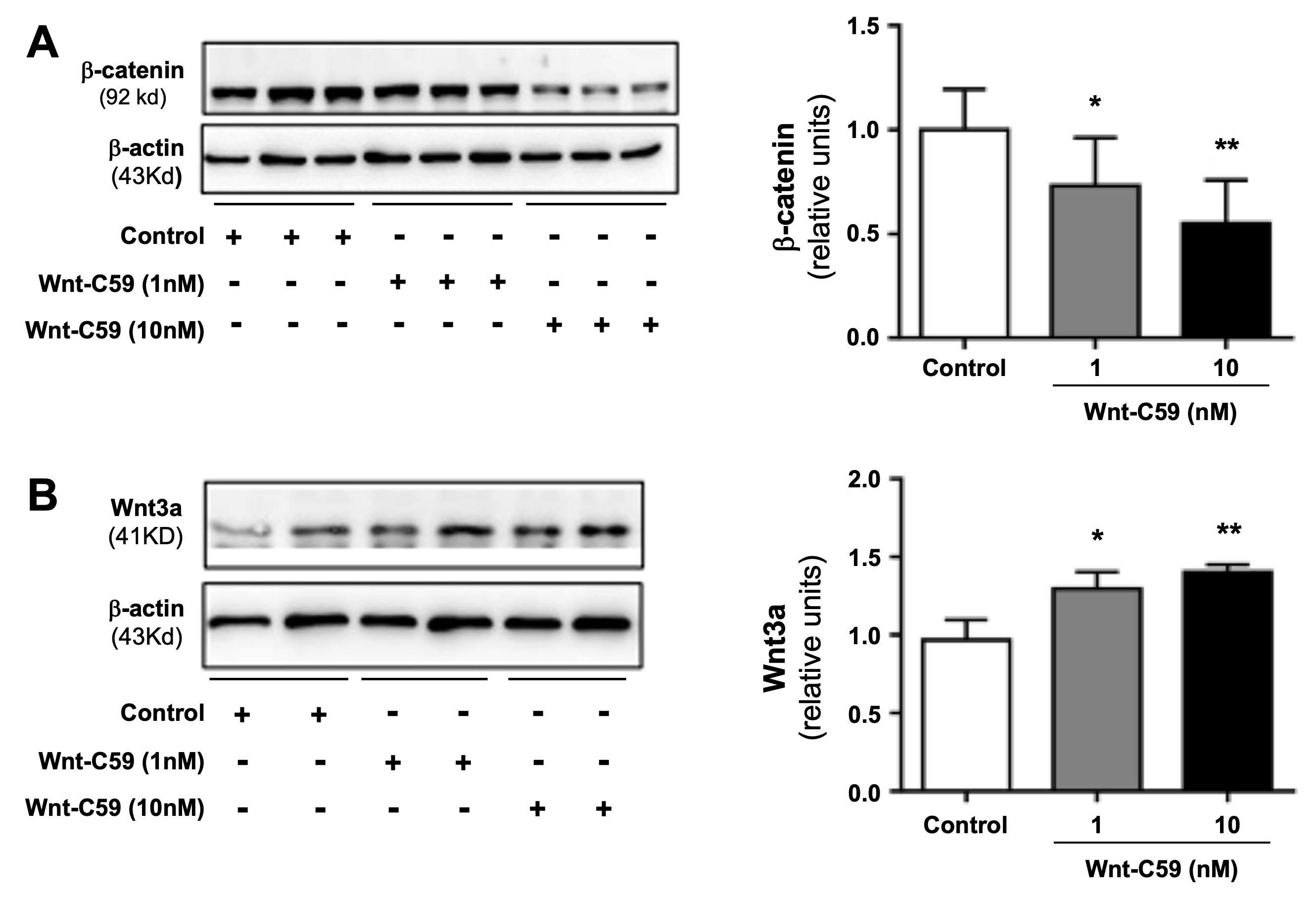

Supplement: Supplementary file 2 — Wnt-C59 prevents accumulation of β-Catenin and increase Wnt3a in L Cells. A. Representative Western blot of β-Catenin levels in L cells that stably express Wnt3a, treated with Wnt-C59 (1 nM and 10 nM C59) for 48 hours. Quantification of β-Catenin protein levels after treatment with Wnt-C59. All data are presented as means ± SEM, it was performed as a two-way ANOVA statistical analysis n = 3. *p <0.05. ** p <0.01. B. Representative Western blot of Wnt3A levels in L cells treated with Wnt-C59 (1 nM and 10 nM) for 48 hours. Quantification of Wnt-3A protein levels after treatment with Wnt-C59 for 48 hours. All data are presented as means ± SEM, it was performed as a two-way ANOVA statistical analysis n = 3. *p <0.05. ** p <0.01. [file 12964_2021_709_MOESM2_ESM.jpg]
